# Menstrual cycle phase and carbohydrate ingestion alter immune response following endurance exercise and high intensity time trial performance test under hot conditions

**DOI:** 10.1186/1550-2783-11-39

**Published:** 2014-08-12

**Authors:** Hideki Hashimoto, Toshimichi Ishijima, Harumi Hayashida, Katsuhiko Suzuki, Mitsuru Higuchi

**Affiliations:** 1 Faculty of Sport Sciences, Waseda University, Saitama, Japan; 2 Products Research & Development Laboratory, Asashi Soft Drinks Co., Ltd, Ibaraki, Japan; 3 School of Human Culture, The University of Shiga Prefecture, Shiga, Japan; 4 Faculty of Health Promotional Sciences, Tokoha University, Shizuoka, Japan

**Keywords:** Prolonged exercise, Leukocytes, Cytokines, Carbohydrate

## Abstract

**Background:**

Sex hormones are known to regulate some responses during exercise. Evaluation of the differences in exercise response with regard to menstrual cycle will help understand the menstrual cycle phase specific adaptations to exercise and athletic performance.

**Methods:**

We investigated the effects of menstrual cycle phase and carbohydrate (CHO) ingestion on immune response during endurance exercise at 30°C. Six healthy women completed 4 trials comprising 90 min of cycling at 50% peak aerobic power $\left(\dot{V}O_{2\mathrm{peak}}\right)$ and a high intensity time trial performance test (POST). They ingested a placebo- or CHO-containing beverage during the trials, which were performed during both the follicular and luteal phases of the menstrual cycle. In all trials, thermoregulatory, cardiorespiratory, and immune responses were measured during exercise and after POST.

**Results:**

Although the thermoregulatory responses differed between the menstrual cycle phases, the cardiorespiratory responses were not different. After placebo ingestion, leukocyte concentration (cells/μL) at POST (15.9 × 10^3^) in the luteal phase was significantly higher than that in the follicular phase (12.9 × 10^3^). The rise in leukocyte concentration was attenuated upon CHO ingestion, and the difference between menstrual cycle phases disappeared. A significant positive correlation was found between leukocyte concentration and serum free fatty acid concentrations. Interleukin-6, calprotectin, and myeloperoxidase concentrations significantly increased at POST in all trials, but no significant differences were observed between menstrual cycle phase or beverage type. Concentrations of other cytokines did not change during exercise in any of the 4 trials. Menstrual cycle phase and beverage type had no significant effect on the POST outcome. Thus, differences in leukocyte mobilization between menstrual cycle phases could result from the effect of sex hormones on substrate utilization.

**Conclusions:**

The menstrual cycle affected circulating leukocyte concentrations during endurance exercise with POST when a placebo was ingested. Therefore, we recommend ingesting CHO beverages to attenuate immune disturbances, especially in the luteal phase, even though they are unlikely to enhance test performance.

## Background

With an increase in the number of women participating in sports for recreation, health, fitness, weight management, social interaction, competition, and/or personal accomplishment, the influence of menstrual cycle phase on physiologic response to exercise has received much attention, not only for athletes but also women in general. Sex hormones are known to regulate substrate utilization [1], muscle fatigue [2], temperature regulation [3], and endocrine response [4] during exercise. Evaluation of the differences in exercise response with regard to menstrual cycle phase will help understand the menstrual cycle phase–specific adaptations to exercise and athletic performance.

A number of studies involving male subjects have reported that exercise causes disturbances in circulating leukocyte concentrations and function and that these effects are dependent on the intensity of exercise and the associated release of stress hormones [5]. Furthermore, body temperature has been shown to affect leukocyte mobilization, cytokines, and markers of neutrophil activation during and after exercise in men. Thus, greater systemic mobilization was observed in a hot environment [6,7].

In women with a normal menstrual cycle, core temperature (Tc) rises by 0.3°–0.5°C in the luteal phase compared to the follicular phase [8]. Therefore, we hypothesized that a stressful condition (higher Tc and cardiorespiratory strain) in the luteal phase would affect immune response to prolonged exercise. Although several studies have reported the effect of exercise on immune response, very few have investigated the effect of menstrual cycle phase. One study reported that menstrual cycle phase did not significantly affect immune cell response (leukocytes, monocytes, neutrophils, and lymphocytes) after 90 min of cycling at 65% maximal aerobic power [9], However, there was no information on Tc during exercise, and it appeared that the concentration of progesterone, which is involved in body temperature regulation, was too low in the luteal phase.

Several studies have investigated the effect of menstrual cycle phase on endurance exercise performance, but consistent results have not been obtained [10,11]. One study showed that endurance performance was significantly decreased during the luteal phase compared with the follicular phase in a hot and humid condition (32°C, 60% relative humidity), even though there was no difference between menstrual cycle phases in a temperate condition (20°C, 45% relative humidity) [12]. One characteristic of that study was to set a strict progesterone concentration (>5.1 ng/mL) as the criterion threshold for definition of the luteal phase.

A number of previous studies involving male subjects reported that CHO ingestion during exercise could suppress the mobilization of leukocytes into the circulation [13-15] and secretion of cytokines [13,15-18]. Ingestion of CHO during prolonged exercise, maintains blood glucose concentration, lessens hypothalamic-pituitary-adrenal activation and diminishes the perturbation of circulating leukocyte concentration and function [19-21].

It is known that ovarian hormones can exert metabolic actions affecting substrate utilization. For example, administration of estradiol and progesterone in rats has been reported to decrease gluconeogenesis from alanine and to increase hepatic storage [22], and variations in plasma ovarian hormones concentrations have been shown to alter gluconeogenesis [23]. Furthermore, animal studies show that larger lipid, and lower CHO, utilization occurs during exercise when estrogen and progesterone, are elevated [24,25]. Exercise substrate utilization in women throughout menstrual cycle has generally been characterized by the measurement of respiratory exchange rate (RER). Although discrepancies occur when studying RER in women at rest or during exercise, some data shows the significant difference between the menstrual cycle phases. Furthermore, previous research shows decrease of blood glucose concentration in luteal phase compared to follicular phase during prolonged exercise [26]. This low blood glucose concentration in the luteal phase may have a different impact on immune response between menstrual phases during prolonged exercise.

The first purpose of our study was to examine the effect of menstrual cycle phase on immune response to exercise in a hot condition, with a progesterone concentration threshold for luteal phase verification. To our knowledge, the interaction between exercise in a hot condition and menstrual cycle phase with progesterone limitation on immune response has not been systematically studied because the exercise conditions in previous studies were not defined. The second purpose of our study was to investigate the effect of CHO ingestion on immune response. We hypothesized that menstrual cycle may affect immune responses and that CHO ingestion attenuates these effects.

## Materials and methods

### Subjects

Six healthy young women volunteered to take part in this study. All subjects maintained a regular menstrual cycle and were not taking any oral contraceptives before testing. Their mean characteristics were as follows: age, 23 (SD, 2.6) years; weight, 48.7 (SD, 6.1) kg; height, 156.2 (SD, 2.4) cm; and peak aerobic power $\left(\dot{V}O_{2\mathrm{peak}}\right)$, 39.5 (SD, 5.3) mL/kg/min. Four subjects had not performed regular physical activities for the previous 3 years, whereas two other subjects performed regular physical activities (e.g. swimming).

### Experimental design

This study comprised 4 separate experimental trials. Subjects exercised in the condition (30 [SD, 2]°C and 50 [SD, 5]% relative humidity) during follicular and luteal phases of their menstrual cycle. During the exercise, subjects either consumed a CHO beverage containing 3.8% CHO (2.1% glucose and 1.7% fructose) or a placebo sweetened with an artificial sweetener (sucralose and acesulfame potassium) that tasted like the CHO beverage. We used a hypotonic CHO beverage (osmolality 195 mOsm/kg), which was reported to attenuate some inflammatory responses to exercise [27]. The components and ingredients of both beverages were otherwise identical. The composition of both beverages was as follows: protein and fat, 0 g/100 mL; sodium, 26 mg/100 mL; potassium, 6 mg/100 mL; calcium, 1 mg/100 mL;, and magnesium, 1 mg/100 mL. Both beverages had the same flavor and color (slightly cloudy) and were served to subjects in a transparent plastic cup. Thus, subjects were blinded as to which beverage they were consuming. Subjects were asked to ingest 300 mL of either beverage 30 min before exercise and another 107 mL every 15 min during the 90-min exercise (7 times), so that the total intake was 1,050 mL per participant. The amount and timing of intake were determined according to the position stands of the American College of Sports Medicine [28]. This study was approved by the Human Research Ethics Committee of the Faculty of Sport Sciences of Waseda University for the use of human subjects in accordance with the Declaration of Helsinki. Prior to participation, each subject provided her informed consent.

### Preliminary testing

To estimate menstrual cycle phase, all subjects recorded their oral temperature upon waking every day for at least 2 months and the day of menstruation for 3 months before commencement of the trial. Additionally, blood samples taken before commencement of the exercise were analyzed for estradiol and progesterone concentrations to determine the menstrual phase.

$\left(\dot{V}O_{2\mathrm{peak}}\right)$ was measured using a maximal graded exercise test with an electromagnetically braked cycle ergometer (Combi RS-232; Combi, Tokyo, Japan). The initial workload was 0 W for 4 min (warming up) and was increased by 30 W every 3 min thereafter, starting at 40 W, until subjects could no longer maintain the required pedaling frequency (70 rpm). Heart rate (HR) was monitored by electrocardiography (Cardiosuper 2E32; Sanei-Sokki, Yamagata, Japan) throughout the exercise. During the progressive exercise test, the expired gas of subjects was collected, and the rates of oxygen consumption $\left(\dot{V}O_{2}\right)$ and carbon dioxide production $\dot{V}CO_{2}$ were measured and averaged over 30-s intervals using an automated breath-by-breath gas analyzer (Minato AE300; Minato Medical Science, Osaka, Japan). $\dot{V}O_{2\mathrm{peak}}$ was defined as the highest 30-s value. At the end of each workload stage, subjects were asked to indicate the rating of perceived exertion (RPE) by using the Borg Scale [29].

### Experimental trials

All subjects completed four separate experimental trials, with each trial occurring at a specific time during the menstrual cycle, previously determined for each subject by her basal body temperature. For the 2 CHO and 2 placebo trials, 1 trial each occurred in the follicular phase (FC and FA, respectively), and 1 trial each in the luteal phase (LC and LA, respectively). To avoid a confounding phase and beverage effect with trial order, subjects were randomly assigned trial orders, with 3 subjects commencing in the follicular phase and 3 in the luteal phase. Each experimental trial was performed on a separate day at least 1 week apart. Subjects were asked to only drink water after 21:00 h on the day before the experimental trial, and they ate a standardized breakfast (protein, 12.4 g; fat, 5.5 g; CHO, 75.7 g; and total energy, 395 kcal) at 06:00 h, i.e. 6–7 h before each trial. Thereafter, foods and beverages, except for water, were not allowed.

In all 4 trials, subjects cycled at 50% $\dot{V}O_{2\mathrm{peak}}$ (60 [SD, 12.2] W) for 90 min in a hot condition (30 [SD, 2]°C and 50 [SD, 5]% relative humidity) and completed POST. The workload corresponding to 50% $\dot{V}O_{2\mathrm{peak}}$ was determined from the graded exercise test by interpolation from the line of the best fit describing the relationship between power output and $\dot{V}O_{2}$. During endurance sports competitions, such as marathons, many participants attempt sprints in the final stage of the race. Therefore, in order to simulate an actual competition, our experimental protocol comprised of 2 parts: 90 min of cycling exercise at moderate intensity and a timed performance test. This study composed of a prolonged exercise and performance test under hot condition by untrained subjects. We set exercise intensity at 50% $\dot{V}O_{2\mathrm{peak}}$, which was lower than the 65% $\dot{V}O_{2\max}$ and room temperature condition set in the previous study [9] because we thought that the 65% $\dot{V}O_{2\max}$ intensity under hot condition would be too strenuous for untrained subjects. To measure rectal temperature during the exercise, subjects self-inserted a rectal probe (401 J; Nikkeiso-YSI Co. Ltd., Musashino, Japan) 10 cm past the anal sphincter. During exercise, minute ventilation $\left(\dot{V}E\right)$, expired gas concentration, HR, and rectal temperature were measured for 3 min at the 4-min (warm-up), 15-min, 30-min, 45-min, 60-min, 75-min, and 90-min time points. Subjects were asked to indicate their overall RPE, RPE-cardiovascular, and RPE-legs to identify specific locations of perceived exertion at every 15-min time point from the warm-up to the end of the 90-min cycling exercise.

Following the 90-min exercise, subjects completed POST that lasted approximately 10 min in the same condition. Subjects were required to complete a set amount of work (52.4 [SD, 8.6] kJ) as fast as possible. The total amount of work to be performed was calculated using the following formula [30]: Total work (J) = 0.65 Wpeak × 600. Wpeak (134.4 [SD, 22] W) was the maximal workload capacity determined in preliminary testing and 600 was the duration in seconds (equivalent to 10 min). The ergometer was connected to a computer that calculated and displayed the total amount of work performed. Subjects received only information on the percentage of work performed relative to the set amount of work from the examiner. A familiarization trial was also completed before commencement to allow subjects to familiarize themselves with the protocol and laboratory setting.

### Blood sampling and analysis

Venous blood samples were collected by venipuncture from an antecubital vein before exercise (PRE); at the 30-min, 60-min, and 90-min time points during the exercise; and at POST. Blood samples were collected into serum separation tubes or vacutainers containing ethylenediaminetetraacetic acid (EDTA). A fraction of whole blood was used to measure hemoglobin, hematocrit, and full blood cell count. Serum separation tubes were left to allow blood to clot at room temperature for 30 min, while vacutainers containing EDTA for plasma separation were immediately centrifuged at 1,000 × *g* for 10 min. Serum and plasma were then removed and stored at -80°C for future analysis. Serum free fatty acid and plasma glucose concentrations, leukocyte concentrations, hemoglobin, and hematocrit were analyzed by BML, Inc. (Tokyo, Japan). Commercial enzyme-linked immunosorbent assay (ELISA) kits were used to measure plasma concentrations of the cytokines interleukin (IL)-1β, IL-1 receptor antagonist (IL-1ra), IL-6, tumor necrosis factor (TNF)-α (R&D Systems, Minneapolis, MN), IL-8, IL-10, and IL-12p40 (Becton Dickinson Bioscience, San Diego, CA), and the neutrophil activation markers myeloperoxidase (MPO) and calprotectin (HyCult Biotechnology, Uden, the Netherlands). ELISA measurements were performed according to the instructions for each ELISA kit using a microplate reader (VERSAmax; Molecular Devices, Sunnyvale, CA). Plasma concentrations of all these variables were adjusted for changes in plasma volume [31].

### Statistical analysis

All data were checked for normal distribution using the Kolmogorov-Smirnov statistic. Data for rectal temperature, HR, VE, RER, blood glucose, serum free fatty acid, leukocyte concentrations, and performance test result were normally distributed. Data for serum sex hormones, IL-8, IL-12p40, and MPO concentrations were normally distributed after log transformation. Data for serum IL-1β, IL-1ra, IL-6, IL-10, calprotectin, and TNF-α concentrations were not normally distributed. Normally distributed data (sex hormones and POST result were analyzed using a 2 × 2 factor (menstrual cycle phase × beverage) repeated analysis of variance (ANOVA). For other normally distributed data (rectal temperature, HR, and RER), a 4 × 8 factor (trial × time) repeated ANOVA was used to determine trial effects, time effects, and trial × time interactions. For other normally distributed data (blood glucose, free fatty acid, leukocyte concentrations, IL-8, IL-12p40, and MPO), a 4 × 5 factor (trial × time) repeated ANOVA was used to determine trial effects, time effects, and trial × time interactions. When significant trial effects, time effects, or trial × time interactions were evident, Bonferroni posthoc multiple comparisons were used. Data for serum IL-1β, IL-1ra, IL-6, IL-10, calprotectin, and TNF-α were analyzed using nonparametric Friedman’s ANOVA on ranks test to determine time effects. Kruskal-Wallis’ one-way ANOVA was used to assess differences between trials at specific time points. Data were analyzed using SPSS version 19 for Windows (IBM Corporation, Armonk, NY) with the threshold for statistical significance set at *P* = 0.05. Relationships between dependent variables and leukocyte concentration were assessed with Pearson product correlations. The level of statistical significance was set at *P* < 0.05.

## Results

### Hormones

Table 1 shows the serum estradiol and progesterone concentrations of all subjects. Menstrual cycle phase × beverage did not affect serum estradiol and progesterone concentrations. A main effect of menstrual cycle phase (*P* < 0.01), but not of the beverage, on both hormones was observed. Serum estradiol and progesterone concentrations were significantly higher in the luteal phase than in the follicular phase [32]. We used the progesterone concentration limit 5.1 ng/mL for luteal phase verification according to previous studies [12,33].

**Table 1:** Resting hormone concentration of subjects before each trial

	**FA**		**LA**		**FC**		**LC**	
**Subject**	**[E] (pg/mL)**	**[P] (ng/mL)**	**[E] (pg/mL)**	**[P] (ng/mL)**	**[E] (pg/mL)**	**[P] (ng/mL)**	**[E] (pg/mL)**	**[P] (ng/mL)**
1	33.4	0.2	171.4	18.9	163.7	0.3	321.3	15.7
2	34.8	0.2	125.5	10.4	42.7	0.1	98.5	7.4
3	30.8	0.4	94.0	6.0	39.4	0.2	68.3	5.7
4	47.3	0.1	108.9	6.4	83.8	1.4	171.0	14.9
5	50.6	0.2	201.4	13.2	98.0	0.2	327.4	19.9
6	201.0	0.4	151.2	25.6	44.0	0.4	102.1	9.1
mean	66.3	0.3	142.1	13.4	78.6	0.4	181.4	12.1
SD	66.5	0.1	40.4	7.0	48.3	0.4	115.7	5.1

*Abbreviations*: *FA* follicular phase placebo trial, *LA* luteal phase placebo trial, *FC* follicular phase carbohydrate trial, *LC* luteal phase carbohydrate trial. [*E*], estradiol concentration, [*P*] progesterone concentration.

### Physiologic response

**Figure 1:**
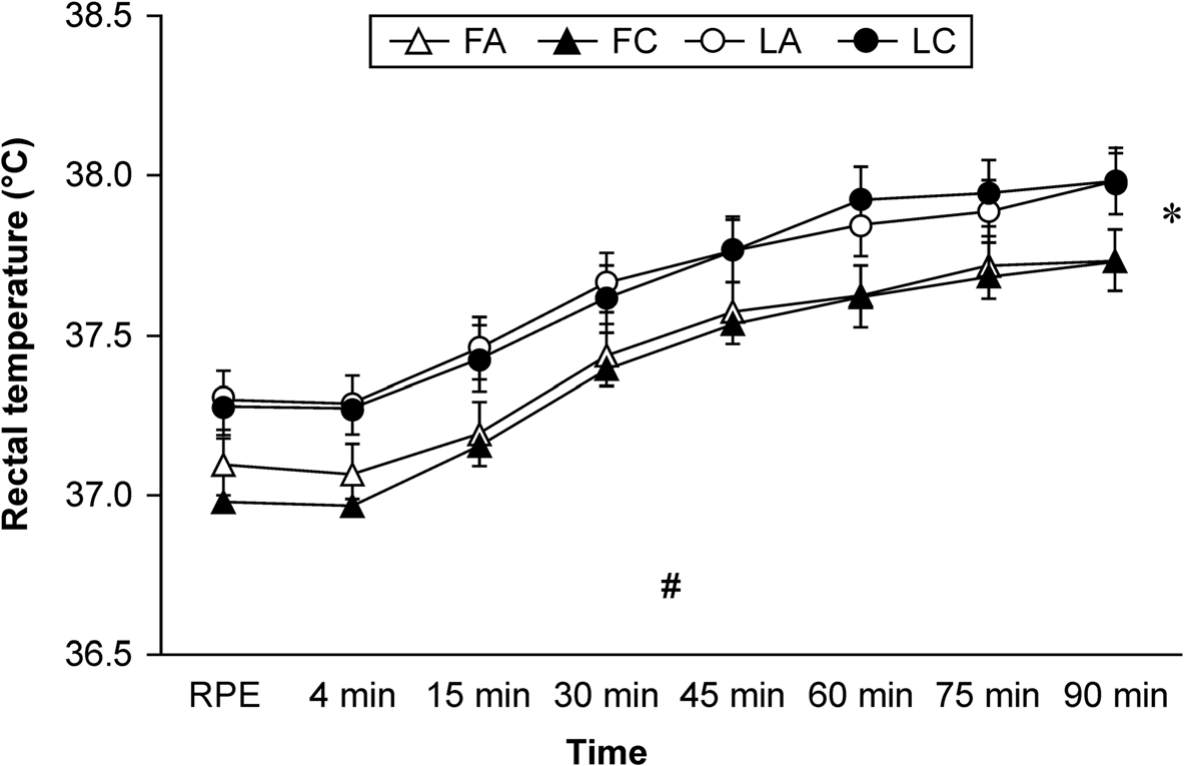
**Rectal temperature during cycling exercise.** Data are mean ± SEM (n = 6). *Significant repeated-measures ANOVA main effect difference between the trials (*P* < 0.05). ^#^Significant repeated-measures ANOVA main effect in time (*P* < 0.01). Abbreviations: FA, follicular phase placebo trial; FC, follicular phase carbohydrate trial; LA, luteal phase placebo trial; LC, luteal phase carbohydrate trial; PRE, before exercise.

Rectal temperature data during exercise is shown in Figure 1.

The results show that menstrual cycle phase and beverage type had significant effect on rectal temperature during exercise (*P* < 0.05). Although there was a significant difference within a trial, there was no significant difference between the trials. Rectal temperature was significantly increased over time during exercise (*P* < 0.01).

**Figure 2:**
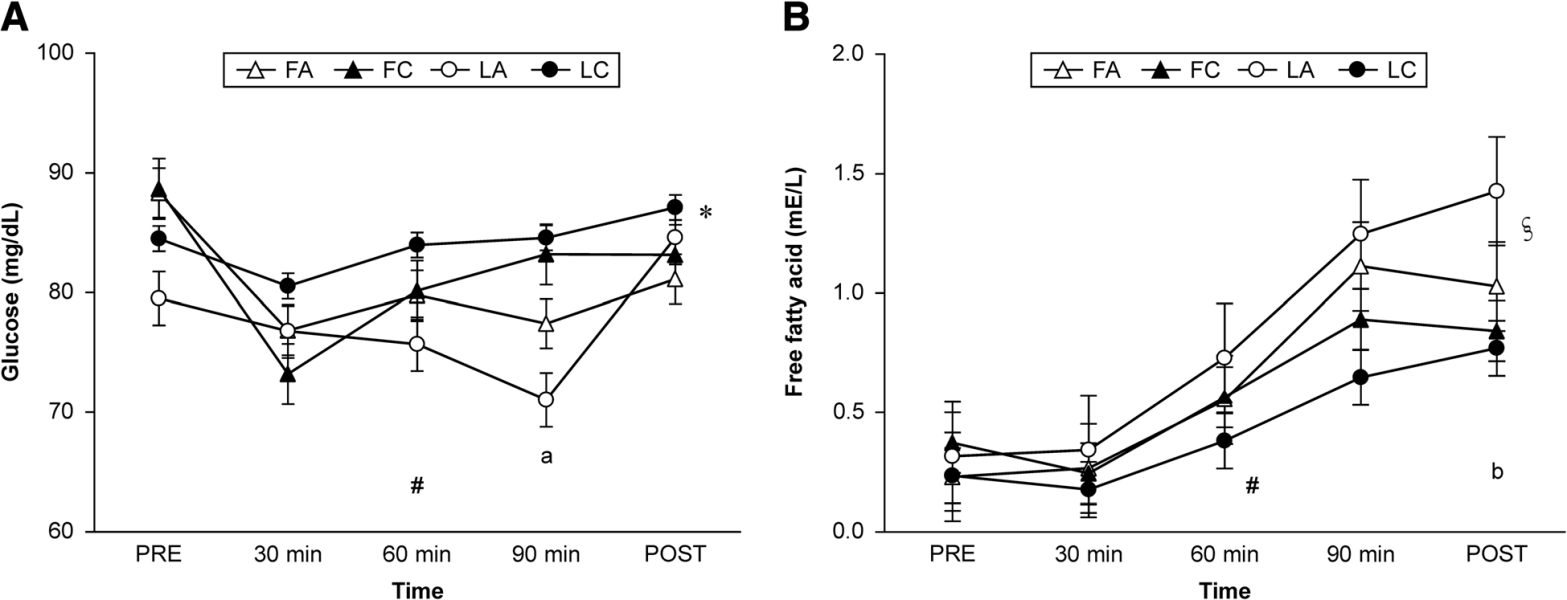
**Blood glucose (A) and serum free fatty acid (B) concentrations.** Data are mean ± SEM. *Significant repeated-measures ANOVA main effect difference between the trials (*P* < 0.05). ^#^Significant repeated-measures ANOVA main effect in time (*P* < 0.01). ^a^Significantly different between LA and LC (*P* < 0.05) ^§^Significant repeated-measures ANOVA interaction between trial and time (*P* < 0.05). ^b^Significantly different between LA and FC (*P* < 0.01). Abbreviations: FA, follicular phase placebo trial; FC, follicular phase carbohydrate trial; LA, luteal phase placebo trial; LC, luteal phase carbohydrate trial; PRE, before exercise; POST, high intensity time trial performance test.

Table 2 shows cardiorespiratory measures. The results show that menstrual cycle and beverage type had no significant effect on HR, $\dot{V}E$, and RER. However HR and $\dot{V}E$ significantly increased to over time during exercise (*P* < 0.01). RER significantly changed over time during exercise (*P* < 0.01). Furthermore, there was no significant effect of menstrual cycle phase or beverage type on RPE data (data not shown).The total amount of CHO consumed in the CHO trials (FC and LC) was 40 g. Plasma glucose and free fatty acid data are shown in Figure 2.

**Table 2:** Cardiorespiratory measures during 90 min exercise

	**Trial**	**4 min**	**15 min**	**30 min**	**45 min**	**60 min**	**75 min**	**90 min**
**HR (beat/min)**	FA	80 ± 50	131 ± 11	138 ± 12	140 ± 12	145 ± 12	149 ± 15	154 ± 15
	FC	83 ± 12	134 ± 14	143 ± 17	145 ± 20	148 ± 19	153 ± 21	156 ± 21
	LA	82 ± 40	137 ± 90	142 ± 11	149 ± 13	148 ± 18	154 ± 13	156 ± 17
	LC	91 ± 16	141 ± 12	149 ± 12	150 ± 12	153 ± 15	154 ± 14	156 ± 14
$\dot{V}E$ **(L/min)**	FA	11.1 ± 1.3	28.4 ± 2.4	29.6 ± 3.4	28.4 ± 4.5	29.8 ± 3.8	29.5 ± 3.3	29.8 ± 3.0
	FC	10.9 ± 1.0	28.7 ± 2.6	29.5 ± 2.3	28.9 ± 3.1	28.6 ± 2.3	30.0 ± 3.3	30.6 ± 3.5
	LA	10.9 ± 2.6	28.9 ± 1.9	30.1 ± 2.4	30.2 ± 4.0	31.0 ± 4.3	32.5 ± 4.9	33.2 ± 6.1
	LC	12.1 ± 1.0	29.1 ± 5.4	30.4 ± 3.4	30.5 ± 4.3	30.4 ± 4.5	30.7 ± 4.5	31.9 ± 4.8
**RER**	FA	0.81 ± 0.04	0.88 ± 0.03	0.88 ± 0.03	0.83 ± 0.05	0.84 ± 0.04	0.82 ± 0.04	0.80 ± 0.04
	FC	0.83 ± 0.08	0.89 ± 0.04	0.87 ± 0.04	0.83 ± 0.04	0.82 ± 0.04	0.82 ± 0.04	0.81 ± 0.04
	LA	0.84 ± 0.05	0.90 ± 0.07	0.89 ± 0.06	0.86 ± 0.08	0.85 ± 0.06	0.85 ± 0.07	0.84 ± 0.08
	LC	0.85 ± 0.11	0.89 ± 0.06	0.88 ± 0.05	0.86 ± 0.04	0.85 ± 0.05	0.84 ± 0.05	0.84 ± 0.05

Dates are shown as mean ± SD. *Abbreviations*: *FA* follicular phase placebo trial, *FC* follicular phase carbohydrate trial, *IL* interleukin, *LA* luteal phase placebo trial, *LC* luteal phase carbohydrate trial, *HR* heart rate, $\dot{V}E$ minute ventilation, *RER* respiratory exchange rate.

There was no trial × time interaction with respect to change in glucose; however, there was a main effect in trial (*P* < 0.05) and time (*P* < 0.01). Blood glucose at 90 min in the LA was significantly lower than that in the LC (*P* < 0.05), and a lower trend compared with that in the FC (*P* < 0.068).

There was a trial × time interaction (*P* < 0.05), a main effect in time (*P* < 0.01), and a trend in trial (*P* = 0.082) in serum free fatty acid. The concentration at POST in the LA was significantly higher compared with that in the FC (*P* < 0.01).

### Immune response

**Figure 3:**
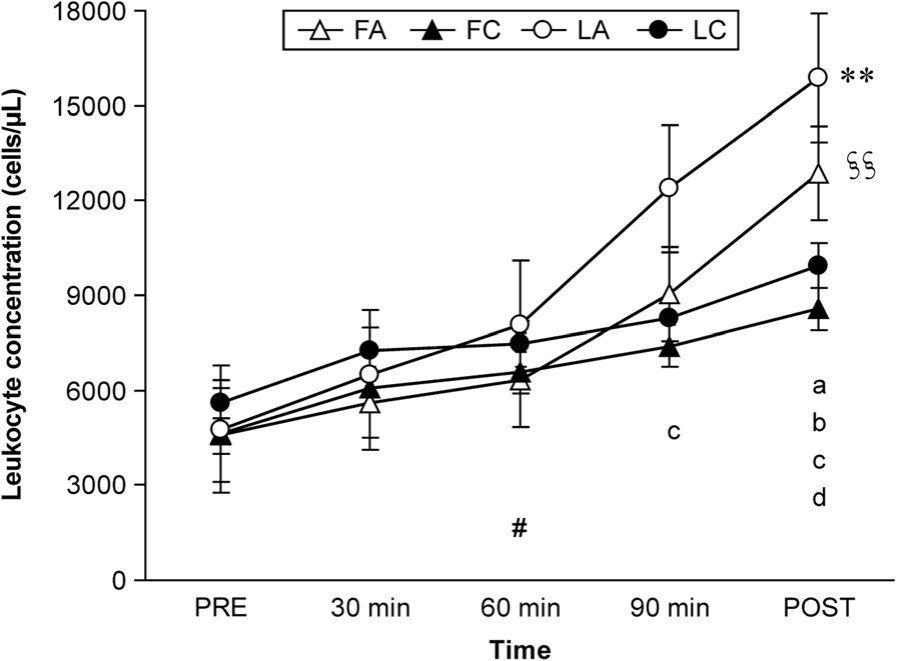
**Leukocyte concentration during exercise and after performance test.** Data are mean ± SEM. **Significant repeated-measures ANOVA a trial × time interaction (*P* < 0.01). ^§§^Significant repeated-measures ANOVA main effect difference between the trials (*P* < 0.01). ^#^Significant repeated-measures ANOVA main effect difference in time (*P* < 0.01).^a^Significantly different between LA and FA (*P* < 0.05). ^b^Significantly different between LA and LC (*P* < 0.01). ^c^Significantly different between LA and FC (*P* < 0.01). ^d^Significantly different between FA and FC (*P* < 0.01). Abbreviations: FA, follicular phase placebo trial; FC, follicular phase carbohydrate trial; LA, luteal phase placebo trial; LC, luteal phase carbohydrate trial; PRE, before exercise; POST, high intensity time trial performance test.

Leukocyte concentration data are shown in Figure 3.

There was a trial × time interaction (*P* < 0.01), and a main effect in trial (*P* < 0.01) and time in leukocyte concentration (*P* < 0.01). Leukocyte concentration at 90 min in the LA was significantly higher than that in the FC (*P* < 0.01), and that at POST in the LA were significantly higher than those in the FA (*P* < 0.05), LC (*P* < 0.01), and FC (*P* < 0.01). Leukocyte concentrations at 90 min in the LA was higher than those in the FA (*P* = 0.073) and LC (*P* = 0.098). Moreover, leukocyte concentrations at POST in the FA were significantly higher than those in the FC (*P* < 0.01). Pearson’s correlation coefficient between serum free fatty acid concentration and leukocyte concentration was determined, and a significant positive correlation was found (*r* = 0.661, *P* < 0.001).Serum cytokine concentration data are shown in Table 3.

**Table 3:** Serum cytokine concentrations

	**PRE**	**30 min**	**60 min**	**90 min**	**POST**
**IL-1β (pg/mL)**					
**FA**	0.9 ± 3.0	0.9 ± 2.5	1.0 ± 3.2	0.9 ± 2.8	0.7 ± 2.9
**FC**	0.9 ± 3.9	1.0 ± 4.9	1.0 ± 3.3	1.2 ± 3.7	1.4 ± 3.3
**LA**	0.9 ± 3.1	1.0 ± 2.8	0.8 ± 2.8	1.1 ± 3.0	1.1 ± 2.8
**LC**	1.2 ± 2.5	1.1 ± 2.7	0.9 ± 2.4	1.1 ± 2.7	1.3 ± 2.2
**IL-1ra (pg/mL)**					
**FA**	186 ± 109	196 ± 131	203 ± 154	256 ± 77	267 ± 130
**FC**	159 ± 59	173 ± 51	234 ± 95	214 ± 121	212 ± 89
**LA**	203 ± 103	200 ± 100	202 ± 83	196 ± 61	269 ± 152
**LC**	234 ± 117	219 ± 138	209 ± 81	224 ± 125	177 ± 115
**IL-6 (pg/mL)**					
**FA**	0.2 ± 1.5	0.1 ± 1.3	0.6 ± 2.3	2.6 ± 3.9^a^	6.3 ± 12.9^b,d^
**FC**	0.1 ± 0.5	0.2 ± 0.3	0.8 ± 1.5	1.9 ± 3.6	2.9 ± 8.8^a,c^
**LA**	0.2 ± 0.2	0.2 ± 0.1	0.5 ± 0.8	1.5 ± 4.0^a^	2.2 ± 9.2^b,d^
**LC**	0.2 ± 0.4	0.2 ± 0.2	0.9 ± 1.2	2.3 ± 2.0^a,c^	4.0 ± 6.6^b,d^
**IL-8 (pg/mL)**					
**FA**	1.2 ± 0.5	1.5 ± 0.5	1.3 ± 0.7	1.7 ± 0.7	2.0 ± 0.9
**FC**	1.5 ± 0.5	1.3 ± 0.5	1.0 ± 0.4	1.5 ± 0.5	1.4 ± 0.6
**LA**	0.8 ± 0.4	1.1 ± 0.4	1.2 ± 0.7	1.2 ± 0.4	1.1 ± 0.5
**LC**	1.1 ± 0.4	1.5 ± 0.6	1.1 ± 0.5	1.6 ± 0.9	1.8 ± 0.7
**IL-10 (pg/mL)**					
**FA**	5.3 ± 4.1	3.8 ± 3.8	5.1 ± 3.8	4.2 ± 1.6	2.8 ± 2.0
**FC**	2.6 ± 2.3	3.0 ± 5.0	3.2 ± 1.3	2.5 ± 2.7	2.9 ± 3.2
**LA**	4.5 ± 8.5	3.3 ± 2.4	3.5 ± 3.2	3.5 ± 3.0	4.9 ± 3.4
**LC**	3.4 ± 3.5	3.7 ± 3.5	3.9 ± 5.6	3.7 ± 2.4	3.4 ± 2.5
**IL-12p40 (pg/mL)**					
**FA**	37 ± 8	44 ± 11	36 ± 6	38 ± 9	43 ± 5
**FC**	29 ± 5	36 ± 4	43 ± 8	27 ± 6	36 ± 6
**LA**	43 ± 7	37 ± 6	32 ± 4	30 ± 10	35 ± 5
**LC**	32 ± 8	32 ± 3	33 ± 3	30 ± 3	34 ± 4
**Calprotectin (ng/mL)**					
**FA**	21 ± 14	26 ± 19	27 ± 24	47 ± 28	95 ± 147^b^
**FC**	18 ± 46	21 ± 121	25 ± 137	39 ± 196^b^	43 ± 267^b,c^
**LA**	23 ± 32	42 ± 29	62 ± 67	170 ± 181^b^	240 ± 117^b,d^
**LC**	26 ± 17	40 ± 45	60 ± 53	64 ± 42^a^	103 ± 276^b,c^
**MPO (ng/mL)**					
**FA**	17 ± 2	20 ± 3	20 ± 1	27 ± 4	46 ± 11^#^
**FC**	20 ± 5	26 ± 8	25 ± 5	38 ± 13	39 ± 9^#^
**LA**	10 ± 2	26 ± 4	24 ± 2	34 ± 3	46 ± 5^#^
**LC**	17 ± 2	21 ± 3	19 ± 3	30 ± 7	34 ± 8^#^
**TNF-α (pg/mL)**					
**FA**	0.3 ± 0.2	0.3 ± 0.1	0.3 ± 0.1	0.3 ± 0.1	0.4 ± 0.2
**FC**	0.3 ± 0.2	0.3 ± 0.2	0.3 ± 0.2	0.3 ± 0.1	0.3 ± 0.1
**LA**	0.3 ± 0.1	0.3 ± 0.1	0.3 ± 0.1	0.3 ± 0.1	0.3 ± 0.1
**LC**	0.3 ± 0.1	0.3 ± 0.1	0.2 ± 0.1	0.3 ± 0.1	0.3 ± 0.1

Data for IL-8, IL-12p40 and MPO are shown as mean ± SEM; data for IL-1β, IL-1ra, IL-6, IL-10, Calprotectin and TNF-α are shown as median ± quartile deviation ^a^Significantly different from PRE (*P* < 0.05). ^b^Significantly different from PRE (*P* < 0.01). ^c^Significantly different from 30 min (*P* < 0.05). ^d^Significantly different from 30 min (*P* < 0.01). ^#^Main effect in time (*P* < 0.01).

*Abbreviations*: *FA* follicular phase placebo trial, *FC* follicular phase carbohydrate trial, *IL* interleukin, *LA* luteal phase placebo trial, *LC* luteal phase carbohydrate trial, *MPO* myeloperoxidase, *POST* high intensity time trial performance test, *PRE* before exercise, *TNF* tumor necrosis factor.

Serum IL-6 concentrations were significantly higher at 90 min (*P* < 0.05) and POST (*P* < 0.01) from PRE in 3 trials (FA, LA, and LC). Moreover, the concentrations at POST in these 3 trials were significantly higher (*P* < 0.01) than those at 30 min. In the FC trial, the concentration at POST was significantly higher compared with that at PRE and 30 min. However, there were no significant differences between the trials at any time point. Serum MPO concentrations were highest at POST in all trials. There was a main effect in time (*P* < 0.01), but no trial × time interaction or main effect in trial. Serum calprotectin concentrations were significantly higher (*P* < 0.01) at POST from PRE in all 4 trials. The concentrations at POST in the FC (*P* < 0.05), LA (*P* < 0.01), and LC (*P* < 0.05) were higher than those at 30 min. Moreover, the concentrations at 90 min in the FC (*P* < 0.01), LA (*P* < 0.01), and LC (*P* < 0.05) were significantly higher than those at PRE.

Serum IL-1β, IL-1ra, IL-8, IL-10, IL-12p40, and TNF-α concentrations remained unchanged following the exercise in all 4 trials, and no significant differences were observed between trials.

### Performance test

The mean time (s) required to complete POST was 675 (SD, 163) in the FA, 681 (SD, 147) in the LA, 692 (SD, 156) in the FC, and 678 (SD, 180) in the LC, respectively. There were no significant differences between trials. Subjects indicated an RPE of 20 for all trials at POST.

## Discussion

The first purpose of the present study was to investigate the effect of menstrual cycle phase on immune response to exercise. The results showed that menstrual cycle phase affected leukocyte concentrations in response to prolonged exercise; leukocyte mobilization in the luteal phase was larger compared to that in the follicular phase at 90 min of cycling and at POST while ingesting a placebo beverage. However, there was no significant effect of menstrual cycle phase on serum cytokine concentrations. Thus, these results partially supported our hypothesis.

The second purpose of this study was to investigate whether CHO ingestion has an effect on immune response to prolonged exercise. The results showed that ingesting a CHO beverage attenuated leukocyte mobilization and eliminated the differences between menstrual cycle phases observed in the placebo beverage trials. However, there was no effect of CHO ingestion on serum cytokine concentrations, which partially supported our hypothesis.

A previous study showed that there was no significant difference in total leukocyte concentration between menstrual cycle phases after 90 min of cycling exercise [9]. The reason for the conflicting results between the previous and present study may be difference in the range of progesterone concentrations (10.3 [SD, 8.3] nmol/L) in the luteal phase. This range indicates that most subjects had a lower progesterone concentration than the 16 nmol/L limit, which may have weakened the effect of progesterone on body temperature [12]. However, the correlation between Tc and immune response could not be determined because no information on Tc was available in the previous study.

It is well known that there is a substantial increase in leukocyte concentration (mainly neutrophils) during endurance exercise, and this increase depends on the intensity and duration of exercise [34]. It has been shown that the elevation of neutrophils is due to several hormones (e.g., epinephrine, cortisol, growth hormone, and prolactin) that are known to have immunomodulatory effects [35]. We hypothesized that higher Tc and potentially increased cardiorespiratory strain in the luteal phase would result in a disturbance in immune response. However, the results of this study showed that there were no significant differences in cardiorespiratory responses between menstrual cycle phases even though thermoregulatory response was significantly different between menstrual cycle phases. Therefore, we thought that causes other than Tc and cardiorespiratory strain could increase leukocyte concentrations in the luteal phase.

Some studies have found that menstrual cycle phase does indeed affect hormonal and metabolic responses to exercise [36,37], particularly in a CHO-depleted nutrition state [26,38]. It is possible that high concentrations of sex hormones in the luteal phase decrease gluconeogenesis and blood glucose concentrations, which, in turn, may lead to an increase in circulating cortisol concentrations and leukocytosis. In this study, the blood glucose concentration at 90 min in the LA trial was lower compared with that in the other trials. Moreover, the high correlation between serum free fatty acid concentration and leukocyte concentration may suggest this mechanism. It is possible that the CHO beverage maintained blood glucose concentrations and prevented the mobilization of stress hormones that cause leukocytosis. Thus, the increase in leukocyte concentration in the LA might be due to the combined effect of exercise and differential substrate metabolism by sex hormones.

Previous studies on male subjects have consistently reported that ingesting CHO during exercise suppresses the rise in circulating neutrophils, most likely by reducing the secretion of stress hormones that regulate neutrophil mobilization by maintaining high blood glucose [13-15]. The results of this study may support these previous studies.

Muscle-derived IL-6 appears to be at least partly responsible for the elevated secretion of cortisol during endurance exercise. Infusion of recombinant human IL-6 into resting humans to mimic the exercise-induced plasma concentrations of IL-6 has been shown to increase plasma cortisol in a similar manner [35,39]. In this study, an increase in IL-6 concentration was observed in all trials, but there were no significant differences between menstrual cycle phases or CHO ingestion at any time point. These results suggest that factors other than IL-6 may be more related to changes in leukocyte concentrations, consistent with a previous study [9].

Most [13,15,17,21,40], but not all, studies [18,41,42] report that carbohydrate ingestion attenuates plasma IL-6 concentration following exercise. The increase in plasma IL-6 concentration observed following all trials in our study could be due to release of IL-6 from the skeletal muscle [43,44]. However, consistent plasma IL-6 concentration between trials was most likely due to consistent release of IL-6 from the skeletal muscle during exercise and suggests that there was no difference in muscle glycogen concentration [45].

Elevated serum MPO concentration after exercise likely reflects neutrophil degranulation because MPO is contained in azurophilic granules within neutrophils. MPO is an important contributor to neutrophil microbicidal activity. Serum MPO concentration depends on exercise intensity [46] and temperature [6]. In the present study, serum MPO concentration was significantly increased at POST compared with PRE, but there were no significant differences between trials. In this study, consistent with other findings [47], CHO intake did not influence change in serum MPO concentration.

Calprotectin is secreted from monocytes and neutrophils in response to a variety of inflammatory conditions [48]. In the present study, serum calprotectin concentration was significantly increased at POST compared with PRE, but there were no significant differences between trials. These results are in agreement with a previous study, in which the effect of heat stress during exercise on change in serum calprotectin concentration was reported [6]. The mechanisms regulating calprotectin release and the biological role of calprotectin during exercise are currently uncertain. We incorporated the POST in the experimental protocol assuming that participants would attempt a sprint similar to that in actual competitions. The study results show that menstrual cycle phase and beverage type had no significant effect on the timed performance test. In this study, cardiorespiratory responses were not significantly different between trials; therefore, no differences in performance between trials could be observed. This result supports a previous study [12], in which menstrual cycle phase did not affect the endurance performance at moderate temperatures.

One of the limitations of this study is the absence of cortisol, growth hormone, catecholamine, and muscle glycogen measurements during exercise, which would have allowed a better understanding of the relationships among hormone concentrations, glucose availability, and differential leukocyte concentrations. Even though we found that menstrual cycle phase significantly affected leukocyte concentration, we cannot rule out the possibility that the small cohort of subjects used in this study might have had a negative impact on some of the other measurements, rendering them non-significant. The small number of subjects may not have provided adequate statistical power to detect real, but relatively small, differences in some of the measured variables.

## Conclusions

Menstrual cycle phase affected circulating leukocyte concentrations during endurance exercise while ingesting a placebo. The degree of leukocyte mobilization was greater in the luteal phase compared with the follicular phase. Ingestion of the CHO beverage eliminated the effect of menstrual cycle phase on leukocyte concentration, which may be explained by the effect of sex hormones on substrate utilization. However, there was no effect of menstrual cycle phase on cytokines during exercise.

The results of this study demonstrate that menstrual cycle changes do alter leukocyte concentration during exercise when subjects consume CHO beverage. Indeed, as the placebo trials progressed, differences became more pronounced, indicating that as endogenous CHO reserves were depleted the effect of menstrual cycle changes become more evident. This was further supported by the absences of difference in immune response between the FC and LC trials.

In women undergoing endurance training for recreational purposes, prolonged exercise in the luteal phase may lead to larger immune disturbances compared to the follicular phase if they ingest water alone during the duration of the exercise. Therefore, we recommend ingesting carbohydrate beverages to attenuate immune disturbances especially in the luteal phase, even though they are unlikely to enhance test performance. However, the total subject number is small and does not constitute a representative sample, and the results need further validation using larger cohorts.

## Abbreviations

CHO: Carbohydrate; POST: high intensity time trial performance test; Tc: core temperature; RER: Respiratory exchange rate; HR: Heart rate; RPE: Rating of perceived exertion; FA: Follicular phase placebo trial; FC: Follicular phase carbohydrate trial; LA: Luteal phase placebo trial; LC: Luteal phase carbohydrate trial; PRE: Before exercise.

## Competing interests

At the time of writing this article, one of the authors was employed at Asahi Soft Drinks Company, which manufactured and supplied the beverage for this study.

## Authors’ contributions

HH, TI, KS, and MH conceived and designed the study. HH, TI, and HH performed the experiments. KS performed blood sampling. HH, TI, and HH carried out immunoassay. HH analyzed the data and wrote the manuscript. All authors read and approved the final manuscript.
